# New insights in the mode of action of anti-leishmanial drugs by using chemical mutagenesis screens coupled to next-generation sequencing

**DOI:** 10.15698/mic2020.02.708

**Published:** 2020-01-21

**Authors:** Arijit Bhattacharya, Sophia Bigot, Prasad Kottayil Padmanabhan, Angana Mukherjee, Adriano Coelho, Philippe Leprohon, Barbara Papadopoulou, Marc Ouellette

**Affiliations:** 1 Dept. of Microbiology, Adamas University, Kolkata, India.; 2 Division of Infectious Disease and Immunity, CHU de Quebec Research Center and Department of Microbiology, Infectious Disease and Immunology, University Laval, Quebec, Canada.; 3 Institute of Biology, University of Campinas, Campinas, Brazil.

**Keywords:** Mut-seq, chemical mutagenesis, resistance, miltefosine, paromomycin, Leishmania

## Abstract

*Leishmania* parasites are responsible for a range of clinical manifestations ranging from self-resolving cutaneous sores to life-threatening diseases. The management of leishmaniasis is complicated in part by the scarcity of treatment options but also by the emerging or established resistance to available drugs. A major driver of resistance in *Leishmania* is the amplification of resistance genes taking advantage of the highly repetitive genomic landscape of the parasite. The recent advent of whole genome gain of function screens gave new momentum to the study of such resistance mechanisms, leading to the identification of novel resistance factors and drug targets against approved drugs, which include antimony (SbIII), miltefosine (MIL), paromomycin (PMM), and amphotericin B. However, these screens do not pinpoint single nucleotide variations (SNVs), an important contributor of drug resistance. To fill the gap, our recent study describes the optimization of chemical mutagenesis coupled to next generation sequencing, an approach called Mut-seq, as a way to explore networks of drug resistance genes in organisms with a diploid to mosaic aneuploid genome like *Leishmania*. Our Mut-seq screen revealed associations between genes linked with lipid metabolism and resistance to MIL, and highlighted the role of a protein kinase in translation leading to resistance to PMM.

Mut-seq **(Fig. 1A)** has been implemented in forward genetic studies for a number of organisms ranging from bacteria to plants. It is optimally advantageous if applied in systems with at least one haploid life-stage. The adaptation of Mut-Seq to *Leishmania* required optimizations in both its design and analysis because of the mosaic aneuploid genome of the parasite with stochastic variations in ploidy and gene copy number during growth. We reasoned that isolating resistant mutants as individual clones on solid medium was essential. Similarly, the use of four different mutagens of varying potency helped increasing mutational diversity and hence the likelihood of generating a sufficient number of resistant mutants. Indeed, analyzing mutants obtained from several independent mutagenesis screens was instrumental to the discovery of gene candidates, as recurrence of SNVs for a given gene between mutants usually meant phenotypic importance. Our approach was to focus first on homozygous mutations and on genes harbouring distinct heterozygous mutations in multiple mutants. We first exploited resistance to MIL as a proof of concept for Mut-seq in *Leishmania.* Indeed, MIL resistance mainly occurs through the acquisition of inactivating mutations in the MIL transporter MT1 (LinJ.13.1590), a phospholipid translocating ATPase. As expected, our Mut-seq screen with MIL revealed MT1 mutations (including several non-sense mutations) in the majority of MIL-resistant mutants, hence validating the approach **(Fig. 1A)**. Mutations in additional genes involved in lipid metabolism were also detected, including SNVs in a fatty acid elongase (LinJ.14.0790), a long chain fatty acyl CoA ligase (LinJ.13.0300) and a glycerol phosphoryl phosphodiesterase (LinJ.36.6220). All contributed to MIL resistance, as validated by a CRISPR-Cas9 based approach to edit the genes in order to incorporate the specific SNVs into otherwise wild-type parasites. These findings clearly implied that lipid translocation and metabolism are crucial for MIL action. We focussed on SNVs found in many mutants, but we identified a plethora of additional mutant-specific mutations, many associated with lipid transport or metabolism, and some of those mutations are likely to be phenotypic as well.

**Figure 1:**
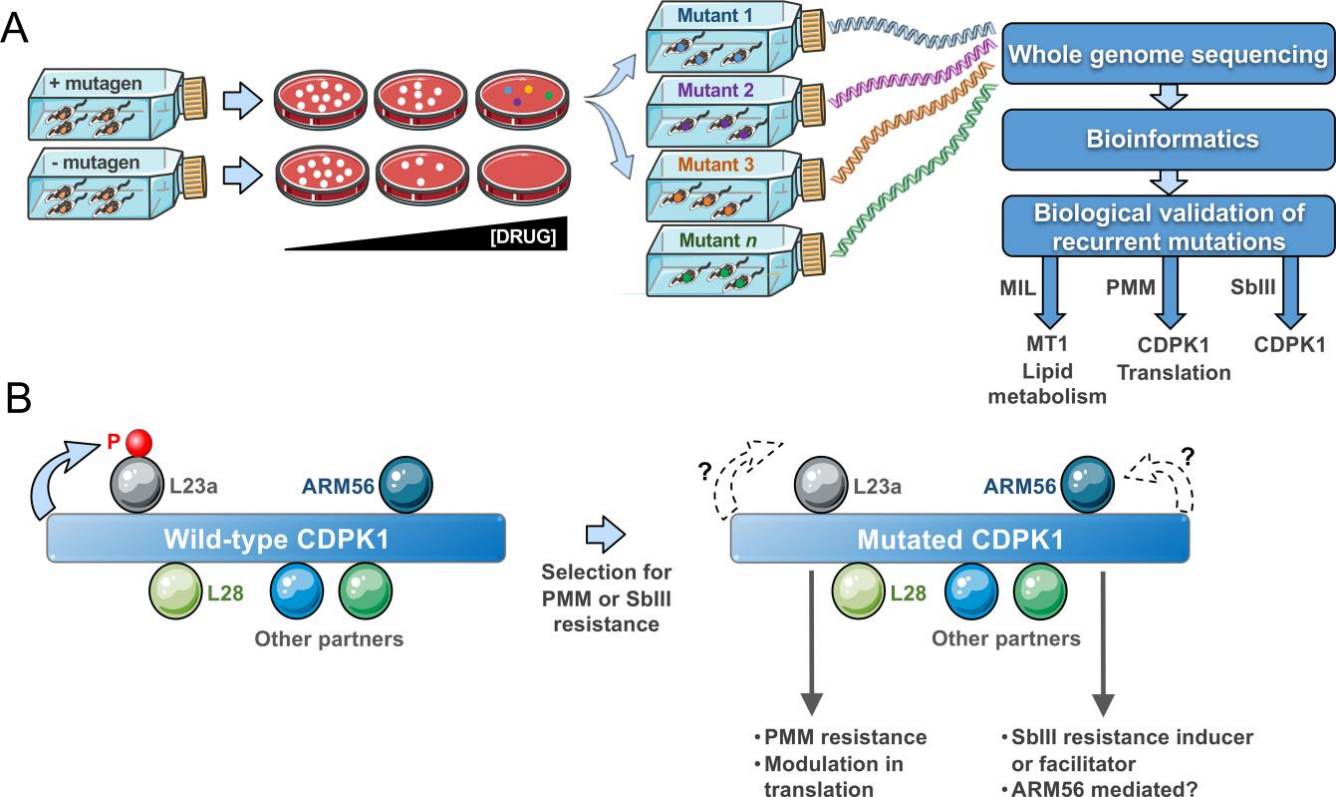
FIGURE 1: A Mut-seq genomic screen highlights the role of the CDPK1 kinase in drug resistance. (A) Mut-seq strategy. Optimisation in mutagens concentrations, recovery time, and plating leads to drug resistant mutant cells. Clones are individually expanded and their DNA sequenced by next-generation sequencing. A bioinformatics pipeline allows the detection of SNVs and recurrent ones are tested experimentally (by transfection and/or gene editing). New genes were isolated using Mut-Seq after selection for miltefosine (MIL), paromomycin (PMM), or antimony (SbIII) resistance. **(B)** CDPK1. Interactome experiments indicated that many partners are potentially interacting with CDPK1. Reciprocal immunoprecipitations confirmed that the ribosomal proteins L23a and L28, and ARM56 a protein associated with SbIII resistance are interacting with CDPK1. L23a is phosphorylated by CDPK1. The Mut-seq screen after selection with either PMM or SbIII revealed that the *CDPK1* gene is frequently mutated. Diverse mutations in CDPK1 were proven to lead to PMM resistance and to modulation in translation. It remains to be seen whether the phosphorylation, or lack of, of L23a is important for those phenotypes. CDPK1 mutations are closely associated with SbIII selection and future work will determine whether its association with ARM56 is necessary for this phenotype. Some of the graphical items in the figure were taken from the Servier Medical Art website and are used under the terms of the Creative Commons Attribution 3.0 Unported License.

The aminoglycoside PMM is in clinical use against leishmaniasis since 2015. In recent years, the structure of the *Leishmania* ribosome has been resolved and interaction with PMM has been elucidated. PMM interacts with the ribosomal proteins S30 and L41 and these were predicted to be hotspots for PMM resistance mutations. Similarly, mutations in rRNA have long been associated with bacterial aminoglycoside resistance including PMM. We have generated several PMM resistant mutants in the laboratory in a step-by-step fashion and the sequencing of their genomes revealed no mutations in either ribosomal proteins or rRNA and as such was not very informative for either resistance mechanism discovery or mode of action of PMM in *Leishmania*. This suggests a significant fitness cost for such mutations and that *Leishmania* probably adopts an alternate strategy to combat PMM-mediated translational arrest. Our Mut-seq screen with PMM, however, highlighted several heterozygous mutations in independent mutants in proteins linked to translation and mRNA stability. We have validated some of these mutations experimentally and showed that they contribute to PMM resistance. A putative calcium dependent protein kinase (LinJ.33.1810), named CDPK1, showed the strongest association with resistance. Several mutations at distinct residues of CDPK1 were found across mutants (including a V366E homozygous mutation) and by episomal overexpression of a wild-type CDPK1 allele in the mutants and by gene editing, we validated these mutations to PMM action. The CDPK1 protein holds a calcium binding domain with a tandem EF-hand motif linked to an N-terminal kinase domain, an organization common in calcium-dependent family of kinases (CDPK) found in plants and Apicomplexan parasites. CDPKs are mostly known as signal transducers impacting multiple cellular functions. However, the *Leishmania* CDPK1 differs from typical CDPKs in bearing an extended C-terminus lacking any identifiable domain and projected to originate from a gene fusion event. The *Leishmania* CDPK family is small with two genes CDPK1 (LinJ.33.1810) and CDPK2 (LinJ.35.0480). CDPK1 exhibits considerable structural conservation with CDPKs from Apicomplexan parasite and it was concluded that it should belong to the category of non-canonical CDPKs. We showed that CDPK1 has kinase activity and is likely to be essential in *L. infantum* promastigotes since we failed to generate a genetic null mutant in the absence of an episomal rescue vector. L-[S^35^]-methionine incorporation experiments and polysome profiling experiments indicated that CDPK1 mutations positively affect ribosomal activity and translation. Such modulation in translation was also observed in cells where one copy of CDPK1 was deleted, suggesting possible interactions between CDPK1 and translation associated proteins and phosphorylation events affecting ribosomal functions. The discovery of putative substrates for CDPK1 was therefore of great interest. A detailed interactome analysis following immunoprecipitations revealed that CDPK1 indeed interacts with a number of proteins related to translation and the unfolded protein response, including the ribosomal proteins L28 and L23a **(Fig. 1B)**. Interestingly, we could establish that the ribosomal protein L23a is phosphorylated by the wild-type version but not by the kinase-dead (i.e. mutated) versions of CDPK1, implying decreased L23a phosphorylation in PMM-resistant mutants. How and whether phosphorylation of L23a impacts on translation rates in PMM-resistant mutants, whether Ca^2+^ can modulate CDPK1 activity, and if its large C-terminus extension contributes to the interactions with the putative cellular partners remain areas to study.

Surprisingly, an independent Mut-Seq screen with selection for SbIII resistance revealed a range of distinct mutations in CDPK1 in the majority of SbIII resistant clones analyzed. These SbIII resistant mutants were cross-resistant to PMM (Bigot *et al.*, unpublished observations). We showed that a single knockout of CDPK1 is more resistant to SbIII and we observed a mutation in CDPK1 in one mutant selected directly for SbIII resistance. However, the exact role of mutations in CDPK1 in SbIII resistance remains to be established. Interestingly, in interactome studies one confirmed CDPK1 partner was ARM56 **(Fig 1B)**, a proven resistance marker for SbIII. Further studies will determine whether ARM56 is indeed phosphorylated by CDPK1 and whether this impacts SbIII resistance. It is thus likely that CDPK1 contributes to PMM and SbIII resistance by different pathways and this is the subject of ongoing studies. More than 30 different mutations have been observed in CDPK1 in both the PMM and SbIII Mut-seq screens. Mutations in CDPK1 were also observed in cells selected step by step for drug resistance to both PMM and SbIII. Such diversity of mutations is intriguing and it remains to be seen whether they are all phenotypic and equivalent. These mutations are loss of function mutations as some mutations are non-sense and a similar phenotype was observed in cells in which the mutation was introduced by gene editing or in cells where one allele was deleted by homologous recombination.

In conclusion, in exploiting Mut-seq we have identified a number of drug resistance/target genes and SNVs in *Leishmania*, a parasite with a mosaic aneuploid genome. We obtained further evidence for the mode of action of MIL and PMM in lipid metabolism and translation, respectively. Additionally, CDPK1 was highlighted as a novel kinase with possibly multiple substrates that may impact diverse cellular functions, including response to at least two drugs. The Mut-seq method might have a broad range application in screening for any selectable phenotype including stress and differentiation conditions to warrant systemic purview of genetic networks associated with diverse biological processes.

